# Altered gut microbiota in older adults with mild cognitive impairment: a case-control study

**DOI:** 10.3389/fnagi.2023.1162057

**Published:** 2023-05-25

**Authors:** Kang-Chen Fan, Chen-Ching Lin, Yi-Chien Liu, Yi-Ping Chao, Yen-Jun Lai, Yen-Ling Chiu, Yi-Fang Chuang

**Affiliations:** ^1^School of Medicine, National Yang Ming Chiao Tung University, Taipei, Taiwan; ^2^Institute of Biomedical Informatics, National Yang Ming Chiao Tung University, Taipei, Taiwan; ^3^Department of Neurology, Cardinal Tien Hospital, New Taipei, Taiwan; ^4^Department of Computer Science and Information Engineering, Chang Gung University, Taoyuan, Taiwan; ^5^Department of Otolaryngology-Head and Neck Surgery, Chang Gung Memorial Hospital at Linkou, Taoyuan, Taiwan; ^6^Division of Medical Imaging, Department of Radiology, Far Eastern Memorial Hospital, New Taipei, Taiwan; ^7^Department of Medical Research, Far Eastern Memorial Hospital, Taipei, Taiwan; ^8^Graduate Program in Biomedical Informatics and Graduate Institute of Medicine, Yuan Ze University, Taoyuan, Taiwan; ^9^Graduate Institute of Clinical Medicine, National Taiwan University, Taipei, Taiwan; ^10^Institute of Public Health, National Yang Ming Chiao Tung University, Taipei, Taiwan; ^11^Department of Psychiatry, Far Eastern Memorial Hospital, New Taipei, Taiwan

**Keywords:** mild cognitive impairment, gut microbiota, 16S ribosomal RNA, cognitive functions, structural brain imaging

## Abstract

**Introduction:**

The microbiota-gut-brain axis is implicated in Alzheimer’s disease. Gut microbiota alterations in mild cognitive impairment (MCI) are inconsistent and remain to be understood. This study aims to investigate the gut microbial composition associated with MCI, cognitive functions, and structural brain differences.

**Methods:**

A nested case-control study was conducted in a community-based prospective cohort where detailed cognitive functions and structural brain images were collected. Thirty-one individuals with MCI were matched to sixty-five cognitively normal controls by age strata, gender, and urban/rural area. Fecal samples were examined using 16S ribosomal RNA (rRNA) V3–V4 sequencing. Compositional differences between the two groups were identified and correlated with the cognitive functions and volumes/thickness of brain structures.

**Results:**

There was no significant difference in alpha and beta diversity between MCIs and cognitively normal older adults. However, the abundance of the genus *Ruminococcus*, *Butyricimonas*, and *Oxalobacter* decreased in MCI patients, while an increased abundance of nine other genera, such as *Flavonifractor*, were found in MCIs. Altered genera discriminated MCI patients well from controls (AUC = 84.0%) and were associated with attention and executive function.

**Conclusion:**

This study provides insights into the role of gut microbiota in the neurodegenerative process.

## 1. Introduction

Mild cognitive impairment (MCI) is a transitional and early-stage cognitive impairment on the continuum of Alzheimer’s disease (AD) (Scheltens et al., 2021). Declines in cognitive performance can take place in various functions, including memory, executive function, attention, language, and visuospatial skills (Albert et al., 2011). Recent findings have suggested that some MCI patients may revert to normal or near-normal cognition, though they are at higher risk of progressing to dementia (Petersen, 2004; Koepsell and Monsell, 2012; Shimada et al., 2019). In other words, MCI is an intermediate phase, and understanding biological changes in this stage may provide insight into mechanisms or intervention targets for delaying the onset of dementia.

The microbiota-gut-brain axis is a complex, bidirectional communication system between the gut and the brain, and several direct and indirect pathways are involved within the axis (Morais et al., 2020). Microbiota is the commensal, symbiotic, and pathogenic microbial communities (bacteria, archaea, fungi, etc.) residing in and on our bodies. Nowadays, the concept of microbiome encompasses microbiota and their “theater of activity,” including structural elements, metabolites, and the surrounding environmental conditions (Berg et al., 2020). The microbiota-gut-brain axis has been implicated in the pathogenesis of AD, and the role of gut microbiota has been beginning to be understood in recent years (Bostanciklioğlu, 2019; Kowalski and Mulak, 2019). Studies in AD have demonstrated a microbial composition that deviates significantly from that of cognitively normal controls (Vogt et al., 2017; Zhuang et al., 2018; Liu et al., 2019; Saji et al., 2019a,b; Guo et al., 2021). There is also emerging data about gut microbiota change in MCI patients (Li et al., 2019; Liu et al., 2019; Saji et al., 2019a,b; Guo et al., 2021; Pan et al., 2021; Sheng et al., 2021, 2022; Zhang et al., 2021; Verhaar et al., 2022; Wanapaisan et al., 2022; Yıldırım et al., 2022). In general, similar gut microbiota changes as AD were found in MCI patients, such as decreased *Bacteroides* genus and increased *Staphylococcus* and *Escherichia* (Li et al., 2019; Nagpal et al., 2019). However, the alterations of gut microbes between studies are far from consistent. This inconsistency may be due to differences in study designs, the study population’s ethnicity, dietary composition, and the criteria used to diagnose AD/MCI. Furthermore, it has been confirmed that there will be significant differences in identifying kinds of gut microbes among analytical pipelines and biobanks (Balvočiute and Huson, 2017; Prodan et al., 2020).

Therefore, we conducted a case-control study in a well-characterized cohort of community-dwelling older adults in Taiwan. We aim to investigate the differences in gut microbiota between older adults with MCI and normal cognition using a more recently-developed and accurate pipeline and biobank.

## 2. Materials and methods

### 2.1. Study design and participants

This case-control study was nested in a dynamic, community-based prospective cohort study, the Epidemiology of Mild Cognitive Impairment in Taiwan (EMCIT) (Chuang et al., 2021). Briefly, the EMCIT recruits independently living adults older than 60 years of age in a rural area and an urban area in North Taiwan with the aim of understanding the epidemiology of MCI. In this cohort, the diagnosis of MCI was adjudicated through the expert panel of a psychiatrist, a neurologist, and a clinical psychologist according to the NIA-AA criteria (Albert et al., 2011). In this study, thirty-one older adults with MCI were matched to sixty-five cognitively normal controls by six strata (age < 75, age > = 75, male, female, urban, and rural areas) between September 2019 to January 2021. The exact numbers of cases and controls in each stratum were presented in Supplementary Table S1. This study was approved by the Far Eastern Memorial Hospital Research Ethics Committee (108058-E) and the Institutional Review Board of the National Yang-Ming University (YM109137E). Informed consent was obtained from all participants.

### 2.2. Fecal sample collection, DNA extraction, and 16S rRNA amplicon sequencing

A fresh fecal sample was collected by participants at the same visit of neuropsychological testing for MCI diagnosis and mailed to Germark Biotechnology (Taichung, Taiwan). Bacterial DNA was extracted using QIAamp Fast DNA Stool Mini Kit (Qiagen, MD, United States). The amount and quality were then determined by NanoDrop ND-1000 spectrophotometer (Thermo Scientific, Wilmington, DE, United States). Extracted DNA was stored at –80°C before 16S rRNA sequencing. V3-V4 regions of bacterial 16S rRNA gene, which are hypervariable, were amplified by PCR using bar-coded primers 341F (forward primer; 5’-CCTACGGGNGGCWGCAG-3′) and 805R (reverse primer; 5’-GACTACHVGGGTATCTAATCC-3′) (Herlemann et al., 2011). Sequencing and library construction of amplicon DNA samples were entrusted to Germark Biotechnology (Taichung, Taiwan). Then, the 2 × 300 bp paired-end amplicon library with an insert size of 465 bp for each sample was prepared using the TruSeq Nano DNA Library Preparation kit (Illumina Inc., San Diego, CA, United States). At last, high-throughput sequencing was performed on an Illumina MiSeq 2000 sequencer with MiSeq Reagent Kit v3 (Illumina).

### 2.3. Microbiome bioinformatic analysis

The demultiplexed 16S rRNA gene sequences generated from the Miseq run were analyzed using the divisive amplicon denoising algorithm 2 (DADA2) pipeline (Callahan et al., 2016). Microbiome FASTQ files were first cleaned by the Cutadapt tool, which removed adapter and primer sequences (Martin, 2011). Subsequently, forward and reverse sequences were truncated at positions 230 and 210, and a maximum number of expected errors were set at 2 and 4, respectively. Error rates were later estimated and corrected by the DADA2 error model parameters. After merging forward and reverse reads to derive full denoised sequences, an amplicon sequence variant (ASV) table was established and disposed of unmatched pairs of reads. Finally, chimeric ASVs were detected and abandoned, and the latest SILVA reference 16S rRNA gene database 138.1 was used to assign taxonomies (Quast et al., 2013).

The resulting ASV table was imported into the Phyloseq package in R to analyze the overall structure of the microbiome, such as phylogenetic tree and biodiversity (McMurdie and Holmes, 2013). Alpha diversity indices, observed OTUs, Chao1 estimator, Shannon, Simpson, and InvSimpson, represented the mean diversity of species in a site within a local scale. The alpha diversity indices between MCIs and controls were compared using linear regression models adjusted for potential confounders. Beta diversity, the change in the diversity of species between the ecosystems, was measured by Jaccard, Bray–Curtis, unweighted UniFrac, and weighted UniFrac distance. The results of these different distance models were plotted by principal coordinate analysis (PCoA). The difference in beta diversity between MCIs and controls would be evaluated by PERMANOVA (permutational multivariate analysis of variance) and Wd*-test (Anderson, 2017; Hamidi et al., 2019). The ASV table underwent a centered-log ratio (CLR) transformation, enabling us to conduct the following compositional analyses (Aitchison, 1982; Badri et al., 2018). To determine differences in genera of interest between MCIs and controls, we performed the linear discriminant analysis (LDA) effect size (LEfSe) method^1^. The LEfSe used Kruskal–Wallis (KW) sum-rank test to detect features with significant differential abundance and a set of unpaired Wilcoxon rank-sum tests to investigate subsequent biological significance among subclasses (Segata et al., 2011). A significant alpha value at 0.05 and an effect size threshold of 2.0 were used to discover genera of interest.

### 2.4. Cognitive assessments

A battery of neuropsychological tests in the Chinese version was used in the EMCIT to assess global cognition and cognitive domains of attention, memory, and executive functions (Chuang et al., 2021). Briefly, global cognition was evaluated by the Mini-Mental State Examination. Tests of attention included the Color Trails Test 1 (CTT 1), subtests of Digit Span (DS), Digit Symbol Substitute Test (DSST), Symbol Search (SS) in the Wechsler Adult Intelligence Scale-III (WAIS-III), and the immediate recall of the Logical Memory subset (LM I) of Wechsler Memory Scale. The delayed recall of the Logical Memory subset (LM II) was used to assess the memory domain. The executive function domain encompassed the Color Trails Test 2 (CTT 2), Semantic Verbal Fluency (VF), and the Stroop Color and Word Test (SCWT). Test scores were all z-transformed, and averaging *z*-scores of tests within each domain generated scores of each cognitive domain.

### 2.5. Brain structure analysis

Every participant was scanned on a 3 T scanner (MAGNETOM Skyra, Siemens Healthcare, Erlangen, Germany) with a 16-channel head coil. The detailed protocol was described elsewhere (Chiu et al., 2019). High-resolution T1-weighted images were acquired using the magnetization-prepared rapid-acquisition gradient echo (MPRAGE) and analyzed using Freesurfer version 7.1.1^2^. Preprocessing, including the skull stripping and reconstruction of the cortical surface, was followed by automated labeling, cortical parcellation, and subcortical segmentation (aparc+aseg). Five MCIs were excluded due to poor image quality, and one normal control was excluded because of brain lesions. Volume data of total brain, ventricles, gray and white matter, and the region of interest (ROIs) that play an important role in AD/MCI, e.g., hippocampus and entorhinal cortex, were obtained. Besides, estimated total intracranial volume (eTIV) would be used to adjust the individual differences in cranial volume.

Furthermore, the average cortical thickness of Alzheimer’s signature MRI biomarkers (Dickerson et al., 2011) was calculated. The Alzheimer-signature brain regions include the medial temporal, temporal pole, inferior temporal, inferior parietal, supramarginal gyrus, superior parietal, precuneus, cauda middle frontal, and superior frontal. The volume of each brain region was obtained by multiplying each region’s thickness by its surface area. Then an AD thickness score was generated by dividing the total volume of the Alzheimer-signature brain regions by the total surface area, representing the mean thickness of Alzheimer-signature brain regions.

### 2.6. Statistical analysis

To examine differences in demographic characteristics, the Pearson chi-square test was applied for categorical variables between groups, while *t*-test was used for continuous variables. Based on results from LEfSe, a group of genera of interest was used to generate a receiver operating characteristic (ROC) curve by pROC R package alone or in combination (Robin et al., 2011). The genera harbored by more than 60% of participants were used further for correlation with cognitive domains and brain structures. Partial correlations between these genera of interest, cognitive domains, and brain structure were adjusted for age, gender, and education years. Correlations were presented in heatmaps by heat function in R. Statistical analyses were done in R version 4.1.2 and Stata 16, and *p* < 0.05 in all tests was considered significant.

## 3. Results

### 3.1. Characteristics of participants

The characteristics of participants between individuals with MCI and cognitively normal controls are presented in Table 1. There were no significant differences in age, gender, and community, the strata of which were used to match the two groups. Participants with MCI had poorer education (6.3 vs. 9.0 years), a higher percentage of hypertension (61 vs. 37%), and a lower frequency of coffee consumption. A total of 8,370,548 non-chimeric high-quality reads were yielded by thirty-one MCIs and sixty-five controls, with an average of 87193.2 reads per sample. On average, these reads for subsequent analysis accounted for 66.8% of raw reads. MCI patients had a slightly lower percentage (65.5 vs. 67.4%)(Supplementary Table S2). A total of 4,925 ASVs were identified.

**Table 1:** Characteristics of the study population.

Characteristics	MCI patients *n* = 31	Healthy controls *n* = 65	*p*-value
Age, years, mean ± SD	73.9 ± 6.7	74.2 ± 6.1	0.83
Gender, male/female	16/15	31/34	0.72
Urban area, *N*(%)	20 (65)	43 (66)	0.87
Education years, mean ± SD	6.3 ± 3.7	9.0 ± 4.4	0.005
Smoking never, *N*(%)	23 (74)	42 (65)	0.35
Drinking never, *N*(%)	20 (65)	41 (63)	0.89
BMI, mean ± SD	24.8 ± 3.1	24.4 ± 3.1	0.48
Diabetes mellitus, *N*(%)	11 (35)	13 (20)	0.10
Hypertension, *N*(%)	19 (61)	24 (37)	0.025
Vegetarian, *N*(%)*			
Vegetarian	2 (6)	5 (8)	0.81
Flexitarian	3 (10)	9 (14)	
Non-vegetarian	26 (84)	51 (78)	
Tea consumption, *N*(%)^†^			
Never	12 (39)	24 (37)	0.29
Sometimes	9 (29)	11 (17)	
Frequently	10 (32)	30 (46)	
Coffee consumption, *N*(%)^†^			
Never	27 (87)	33 (51)	0.002
Sometimes	3 (10)	11 (17)	
Frequently	1 (3)	21 (32)	
Neuropsychological test score, mean ± SD			
MMSE	24.6 ± 3.1	27.5 ± 2.1	<0.001
LMII	5.4 ± 5.1	14.7 ± 7.9	<0.001
LMI	15.1 ± 6.4	27.9 ± 11.1	<0.001
DS	13.8 ± 4.2	17.1 ± 4.1	<0.001
DSST	31.1 ± 17.2	47.9 ± 19.5	<0.001
SS	14.0 ± 7.1	23.8 ± 8.5	<0.001
CTT1 (sec)	115.1 ± 78.4	67.0 ± 36.9	<0.001
VF	30.0 ± 8.2	36.6 ± 8.4	<0.001
CTT2 (sec)	228.1 ± 111.0	142.2 ± 69.9	<0.001
SCWT-color	55.3 ± 12.1	72.6 ± 18.8	<0.001
SCWT-word	65.0 ± 17.2	86.0 ± 21.1	<0.001
SCWT-colored word	28.3 ± 10.7	41.7 ± 15.6	<0.001
SCWT-interference	−1.9 ± 8.6	2.6 ± 11.6	0.11
Memory	−0.8 ± 0.6	0.3 ± 0.9	<0.001
Attention	−0.6 ± 0.7	0.3 ± 0.7	<0.001
Executive functions	−0.5 ± 0.7	0.2 ± 0.6	<0.001

BMI, body max index; MMSE, Mini-Mental State Examination; LM, Logical Memory Test; DS, Digit Span; DSST, Digit Symbol Substitute Test; SS, Symbol Search; CTT, Color Trails Test; VF, Semantic Verbal Fluency; SCWT, Stroop Color and Word Test. *Vegetarian, a person who does not eat meat or meat products; Flexitarian, a person who follows a vegetarian diet but occasionally eats meat or fish; Non-vegetarian, a person who eats meat. ^†^Never, less than 1 time per week; Sometimes, more than 1 but less than 5 times per week; Frequently, more than 5 times per week.

### 3.2. Biodiversities: alpha and beta diversity

**Figure 1:**
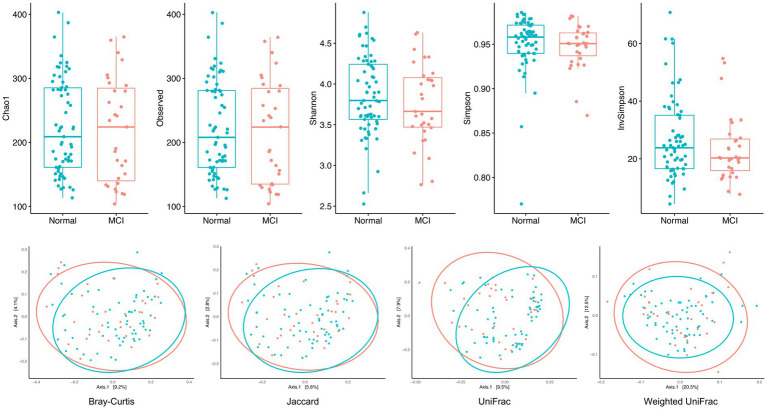
Alpha and beta diversity in MCI patients and healthy controls. Alpha diversity and richness indices (observed OTUs, Chao1 estimator, Shannon, Simpson, and InvSimpson) were both similar between the two groups, representing similar biodiversity within each sample. The beta diversity presented by PCoA (measured by Jaccard, Bray–Curtis, unweighted UniFrac, and weighted UniFrac distance) was also similar, which means that similarities and dissimilarities between samples were indistinguishable.

Figure 1 shows the biodiversity comparisons in MCIs and controls. Alpha diversity and richness were both similar between the two groups, representing similar biodiversity within each sample. After adjusting for age, gender, rural/urban area, education, and hypertension, the indices of the MCI group remained lower than those of controls, although not statistically significant (Supplementary Table S3). The beta diversity presented by PCoA was also similar, which means that similarities and dissimilarities between samples were indistinguishable. Only unweighted Unifrac distance indicated that there was a differential clustering pattern by PERMANOVA and Wd* test (*p*-value < 0.05) (Supplementary Table S4). In summary, the alpha and beta diversity analyses did not show significant differences between individuals with MCI and cognitively normal controls.

### 3.3. The compositional differences between MCIs and controls

**Figure 2:**
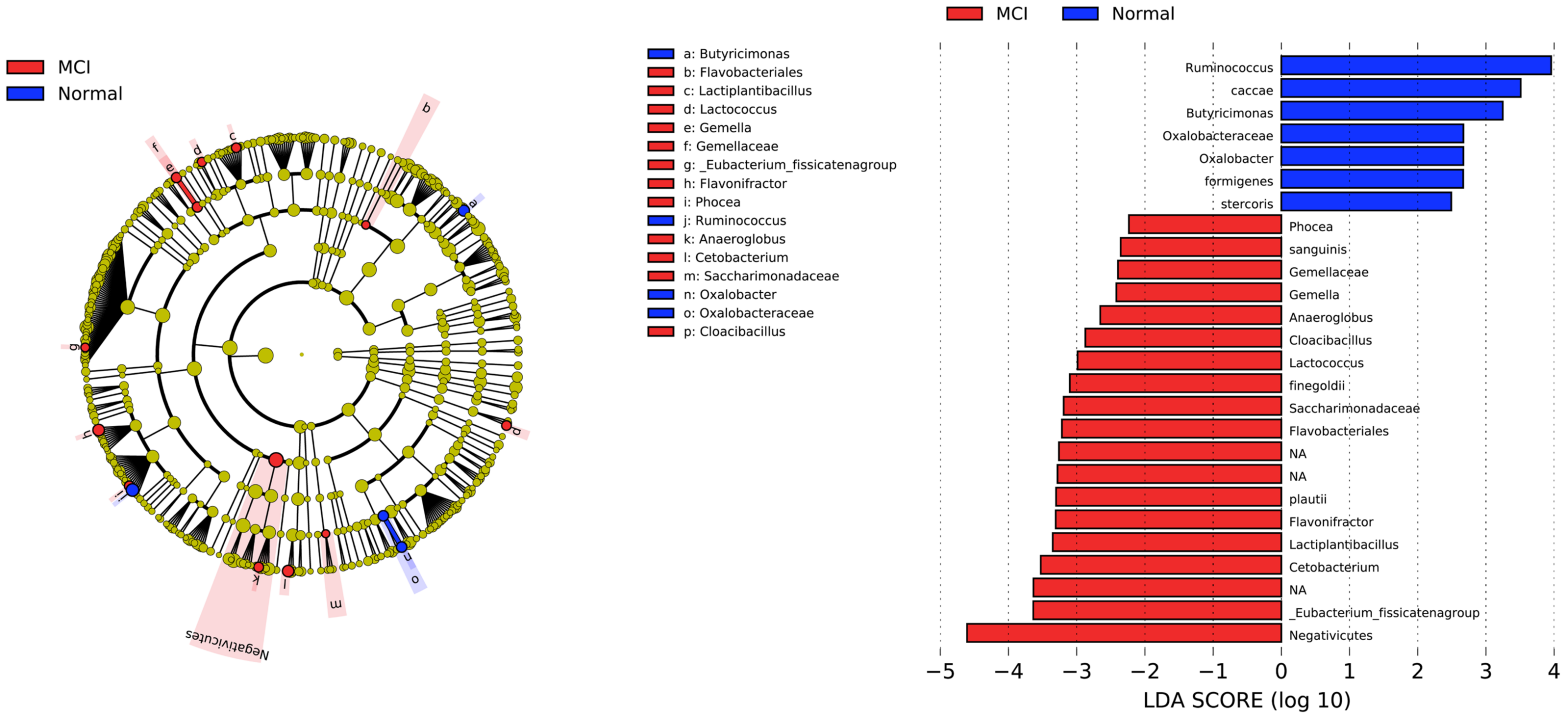
Differential bacterial taxonomies in LEfSe. The LDA barplot and cladogram indicated the differentially abundant microbiota between MCIs and controls (LDA score > 2, *p* < 0.05). At the order level, two bacterial orders, including *Negativicutes* and *Flavobacteriales*, were significantly abundant in individuals with MCI. We also found that at the family level, *Gemellaceae* and *Saccharimonadaceae* had increased in the MCI group, while *Oxalobacteraceae* were more abundant in controls. Twelve genera of interest varied between the two groups. Nine genera: *Phocea*, *Gemella*, *Anaeroglobus*, *Cloacibacillus*, *Lactococcus*, *Flavonifractor*, *Lactiplantibacillus*, *Cetobacterium*, *Eubacterium fissicatena group* had increased in MCI, while three genera, *Ruminococcus*, *Butyricimonas*, and *Oxalobacter*, had increased in controls.

The compositional analysis identified taxa of interest that differed between individuals with MCI and normal controls (LDA score > 2, *p* < 0.05, Figure 2). The LDA barplot and cladogram indicated the differentially abundant microbiota between the two groups. At the order level, two bacterial orders, including *Negativicutes* and *Flavobacteriales*, were significantly abundant in individuals with MCI. We also found that at the family level, *Gemellaceae* and *Saccharimonadaceae* had increased in the MCI group, while *Oxalobacteraceae* were more abundant in controls. Twelve genera of interest varied between the two groups. Nine genera: *Phocea*, *Gemella*, *Anaeroglobus*, *Cloacibacillus*, *Lactococcus*, *Flavonifractor*, *Lactiplantibacillus*, *Cetobacterium*, *Eubacterium fissicatena group* had increased in MCI, termed as MCI-abundant genera in the subsequent analysis. Three genera, *Ruminococcus*, *Butyricimonas*, and *Oxalobacter*, had increased in controls and were called control-abundant genera. Finally, as shown in Figure 2, several species were found to be different between the two groups; however, species identification is more prone to inaccuracy in 16S rRNA analysis. CLR transformed relative abundances of twelve genera different between MCIs and controls were shown in Supplementary Figure S1.

**Figure 3:**
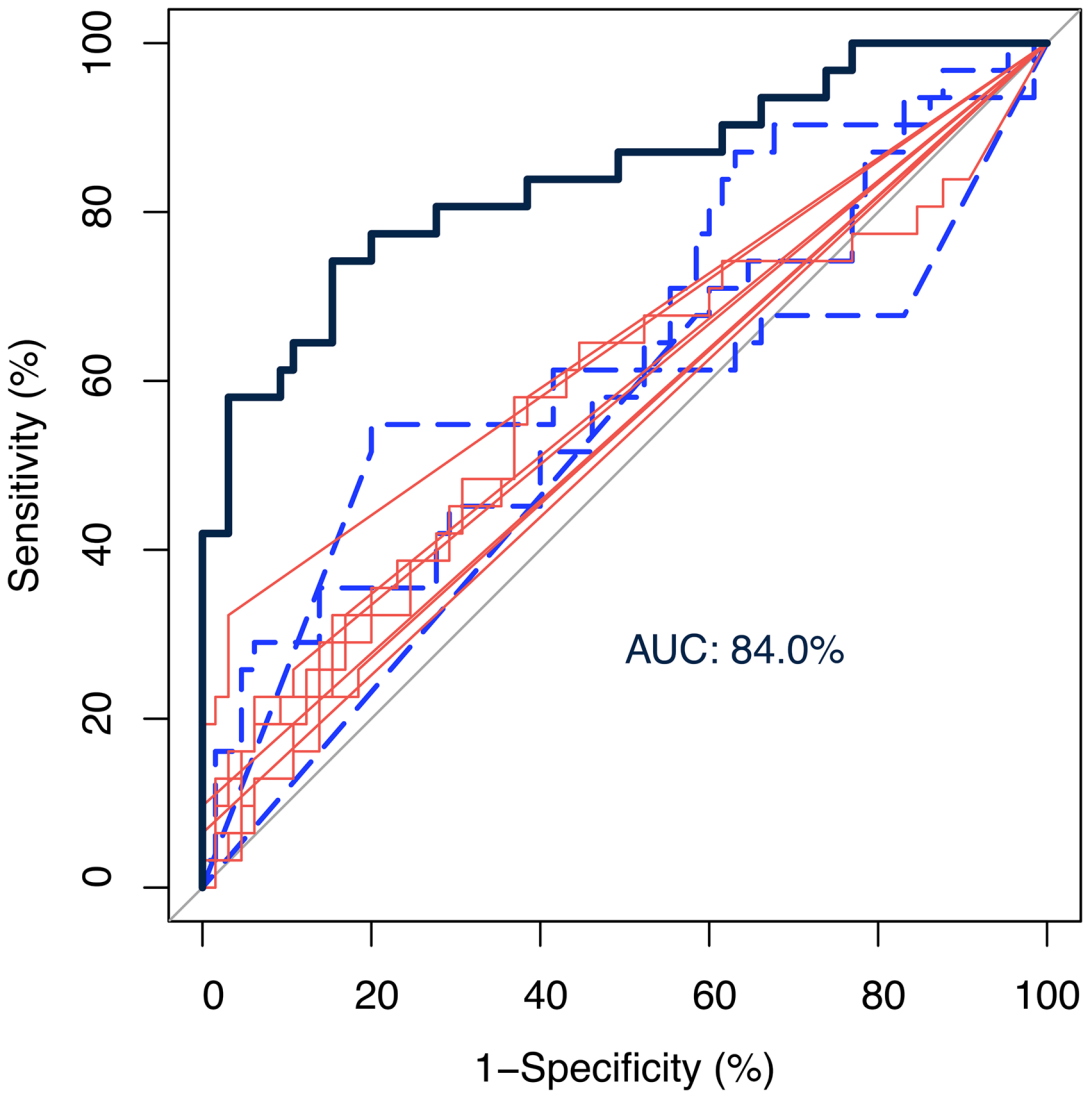
Receiver operating characteristic (ROC) curves of genera as biomarkers. The ROC curves were fitted by the centered-log ratio transformed value of twelve genera of interest alone and all together. The blue-dashed and red-solid curves represented control-abundant and MCI-abundant genera, respectively. The black curve was fitted by logistic regression incorporating all twelve genera of interest as predictors. The twelve genera altogether showed a good performance in discriminating MCI from control [area under the ROC curve (AUC): 84.0%], demonstrating the potential of gut microbiota as a classification model.

Figure 3 shows the ROC curves using the centered-log ratio transformed value of twelve genera of interest alone and all together. The blue-dashed and red-solid curves represented control-abundant and MCI-abundant genera, respectively. The black curve was fitted by logistic regression incorporating all twelve genera of interest as predictors. The twelve genera altogether showed a good performance in discriminating MCI from control [area under the ROC curve (AUC): 84.0%], demonstrating the potential of gut microbiota as a classification model.

### 3.4. Correlations between genera of interest and cognitive domains and brain structures

**Figure 4:**
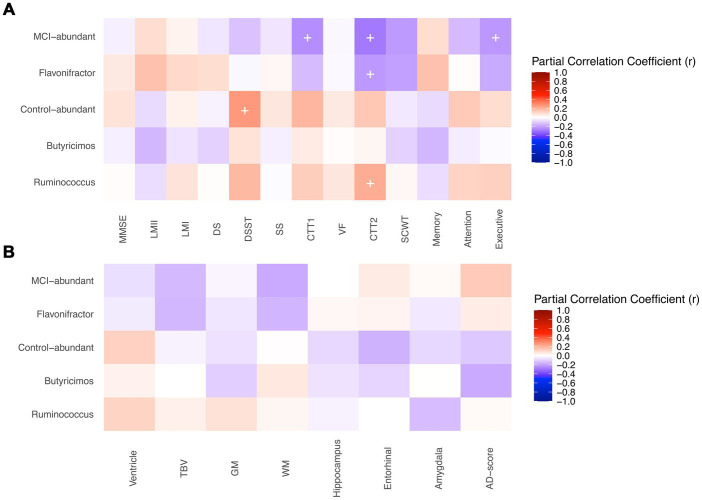
Correlations between genera of interest and **(A)** cognitive tests **(B)** brain structure. MMSE, Mini-Mental State Examination; LM, Logical Memory Test; DS, Digit Span; DSST, Digit Symbol Substitute Test; SS, Symbol Search; CTT, Color Trails Test; VF, Semantic Verbal Fluency; SCWT, Stroop Color and Word Test interference score; TBV, Total Brain Volume; GM, Gray Matter Volume; WM, White Matter Volume. + *p*-value < 0.05. Partial correlations between the genera of interest and the cognitive functions and volumes/thickness of brain structures were shown. **(A)** Partial correlations of genera of interest and neuropsychological tests’ scores were adjusted for age, gender, and year of education. Outcomes of color trails test 1 and 2 were added a minus sign to make the colors of correlations indicate the same direction. The MCI-abundant genus, *Flavonifractor*, was associated with poorer performance in Color Trails Test 2. Moreover, these nine MCI-abundant genera collectively had a significant negative association with executive function and Color Trails Test 1 and 2. In contrast, control-abundant genera altogether were associated with better performance in Digit Symbol Substitute Test. *Ruminococcus* was positively correlated with Color Trails Test 2**. (B)** Partial correlations genera of interest and brain volume indicators (cm^3^) were adjusted for age, gender, and estimated intracranial volume (eTIV). Correlations of genera and AD-score, a cortical thickness indicator (cm), were only adjusted for age and gender. Ventricle included lateral ventricle, lateral inferior ventricle, and 3rd, 4th, 5th ventricles. The genera of interest did not show significant associations with the volumes/thickness of brain structures.

Among twelve genera of interest, some were present in very few individuals, and it’s inappropriate to correlate their abundance with other clinical features because of scarcity. As a result, only genera harbored by more than 60% of participants were included in correlation analysis, including *Flavonifractor*, *Butyricimos*, and *Ruminococcus*. Figure 4 demonstrates the partial correlations between the abundance of the genera above and the cognitive functions and volumes/thickness of brain structures. The MCI-abundant genus, *Flavonifractor*, was associated with poorer performance in Color Trails Test 2 (*r* = −0.22, *p*-value = 0.04). Moreover, these nine MCI-abundant genera collectively had a significant negative association with executive function (*r* = −0.22, *p*-value = 0.03) and Color Trails Test 1 (*r* = −0.24, *p*-value = 0.02) and 2 (*r* = −0.29, *p*-value = 0.005). In contrast, control-abundant genera altogether were associated with better performance in Digit Symbol Substitute Test (*r* = 0.26, *p*-value = 0.01). *Ruminococcus* was positively correlated with Color Trails Test 2 (*r* = 0.21, *p*-value = 0.047).

The volumes/thickness of brain structures between the groups is shown in Supplementary Table S5. Cognitively normal adults have generally larger total brain and gray/white matter volume, as well as the hippocampus, entorhinal, and amygdala, though not significantly different. AD-score thickness is identical between groups. The genera of interest did not show significant associations with the volumes/thickness of brain structures (Figure 4B).

## 4. Discussion

In this case-control study nested in a well-characterized community-based cohort, altered gut microbiota was found in individuals with MCI. Twelve genera of gut microbiota had a good performance differentiating MCI from cognitively normal controls. MCI-abundant genera such as *Flavonifractor* were associated with poorer executive functions, whereas genera more abundant in controls, especially *Ruminococcus*, were linked to better performance.

The finding that no differences in biodiversity of gut microbiota were observed between individuals with MCI and normal cognition is consistent with previous studies (Li et al., 2019; Liu et al., 2019, 2021; Nagpal et al., 2019; Zhang et al., 2021; Hung et al., 2022). On the contrary, the reported microbial compositional differences between MCI patients and healthy controls varied considerably (Liu et al., 2019, 2021; Saji et al., 2019a; Guo et al., 2021; Pan et al., 2021; Zhang et al., 2021). We also revealed other families or genera of gut microbiota associated with MCI. Discrepancies may come from methodological differences, including the source of participants (memory clinic or community), the criteria used to diagnose MCI, and pipelines used to analyze gut microbiota. Most studies published before 2022 (Li et al., 2019; Liu et al., 2019, 2021; Guo et al., 2021; Pan et al., 2021; Sheng et al., 2021; Zhang et al., 2021) relied on OTU-or ZOTU-picking strategies and annotations in QIIME, which is more error-prone than DADA2-denoised ASV tables (Prodan et al., 2020). Therefore, different analytical pipelines coupled with various databases (SILVA or Greengene) used in each study made the results hardly comparable. Furthermore, since microbiota could be heavily dependent on the living environment and dietary styles, our study results are more likely to be comparable to studies conducted in Asia, including China, Japan, and Thailand instead of those in the Netherlands and Turkey.

We found that the genus *Ruminococcus*, *Butyricimonas*, and *Oxalobacter* decreased in participants with MCI, and the abundance of the genera was associated with better attention and executive function. *Ruminococcus* spp., one of humans’ most abundant flora, plays a crucial role in deconstructing, fermenting, and utilizing a wide range of dietary plant polysaccharides into various nutrients and finally affects human health status (la Reau and Suen, 2018). Polysaccharides have long been seen as a modulator in the gut-brain axis. One of the significant components of dietary polysaccharides reaching the gut is insoluble plant fibers, or called dietary fibers, such as inulin, fructooligosaccharides, and pectin (Sun et al., 2023). These non-starch polysaccharides (NSPs) are rich in high-fiber diets, e.g., traditional rural African diets (De Filippo et al., 2010; Ho Do et al., 2021). Moreover, an animal study discovered that *Ruminococcus gnauvus* monocolonized mice performed better on a spatial working memory test and were associated with metabolites such as tryptamine, indolacetate, and TMAO (trimethylamine N-oxide). Tryptamine induces serotonin release through enterochromaffin cells, while indole-derived metabolites regulate the increase of the hippocampus’s neural progenitor cells, some showing anti-inflammatory effects on the brain. Furthermore, TMAO has protective effects on BBB integrity (Hoyles et al., 2021; Coletto et al., 2022). The genus *Butyricimonas* produce butyrate, one of the short-chain fatty acids shown to exert crucial cognitive-protecting effects on the central nervous system. Butyrate prevents cognitive decline by improving barrier function and acting as an assumed negative regulator of amyloidosis and neuroinflammation (Marizzoni et al., 2020). In addition, *Butyricimonas* are grouped into taxa that increase with age yet deplete in unhealthy aging (Ghosh et al., 2022). Finally, *Oxalobacteraceae*, *Oxalobacter*, and *Oxalobacter formigenes* found in this study are less discussed in gut-brain communications. Some research highlighted the pathogenic role of oxalate distribution in the entorhinal cortex; nevertheless, the potential effect of oxalate on cognitive decline remains unclear (Heller et al., 2020).

In our study, several taxa found to increase in MCI patients were reported to increase in the AD patients. These taxa include the genus *Anaeroglobus*, the family *Gemellaceae*, and the genus *Gemella*. In addition, the genus *Flavonifractor* (Coello et al., 2019; Ogita et al., 2020) and the *Eubacterium fissicatena group* (Heo et al., 2016; Xiao et al., 2021) enriched in MCI in our study may involve an escalation of inflammation, oxidative stress, and proinflammatory response. *Flavonifractor* was further correlated with poor performance in the executive function test in our study. Derailed systemic immune system via circulating cytokines and increased gut inflammation is one of the proposed pathways linking the gut microbiota and the brain (Cattaneo et al., 2017). Intriguingly, *Lactococcus* and *Lactiplantibacillus*, both lactate-producing and commonly used as probiotics, produce several metabolites in the nervous system, such as serotonin, acetylcholine, histamine, and dopamine (Alkasir et al., 2017), which were increased in MCI patients. The increased amount of these two genera in MCI patients may imply the compensatory response toward the overrepresentation of inflammation-producing gut microbiota.

Some studies examined the cross-sectional relationship between gut microbiota and brain imaging. *Akkermansia*, *Lachnospiraceae* NK4A136 group spp., and *Anaerostipes* spp. were found to be correlated with medial temporal atrophy or global cortical atrophy (Li et al., 2019; Verhaar et al., 2022). MCI patients with more *Bacteroides* are more likely to present brain atrophy patterns compatible with AD (Saji et al., 2019a). Regarding regional brain volume, Wanapaisan et al. have discovered associations of left and right-hippocampus and right amygdala volumes with groups of bacteria identified in their study (Wanapaisan et al., 2022). However, they also enrolled AD patients and mainly compared brain volumes in controls with AD/MCI. The insignificant correlations between brain volumes/thickness and the relative abundance of microbiota in our study may be explained by the early stage of cognitive impairment and less brain volume change in the individuals with MCI from the community.

Our research had several strengths. First, the study population was derived from a well-characterized community-based cohort, which better represents older adults’ lifestyles and characteristics in the community than those from clinics. In addition, the diagnosis of MCI was rigorously made through a panel of experts. Second, we used the state-of-the-art pipeline with the best sensitivity and accuracy among all the 16S rRNA pipelines. The database was also the latest updated version. Third, we corroborated the results of identified bacteria by examining the relationship with other outcome assessments, cognitive functions, and brain volumes/thickness. This study is not without limitations. First, a single fecal sample was collected from each participant, rendering temporality hard to infer because microbial composition possibly fluctuates within a person over time. More importantly, 16S rRNA sequencing gives us a picture of bacterial composition; nevertheless, it fails to pinpoint species precisely with a complete view of microbiota. Moreover, we did not assess metabolites, pro-inflammatory markers, and BBB integrity. Hence, we could only postulate mechanisms but not construct robust associations. Second, although covariates were carefully measured and used for adjustment in analysis, unmeasured and residual confounding may still exist. For example, adjusting for levels of educational attainment in the analysis of cognitive functions may not fully account for confounding effects of factors closely related to the education level, such as healthy lifestyles diet, or socioeconomic resources. These factors could directly impact the gut microbiome or indirectly by modulating other behaviors (Herd et al., 2018). To what extent these factors confound the relationship between gut microbiota and cognitive function should be addressed in future research. Finally, although our sample size for gut microbiota analysis is comparable with other studies, it could still be insufficient to detect the relationship between the identified genera with subtle differences in brain volume/thickness. Future research with a larger sample size is needed to elucidate the brain structural differences associated with gut microbiota.

In conclusion, MCI was associated with altered gut microbiota, which further correlated with the performance of attention and executive functions. This altered gut microbiota collectively can differentiate MCI from cognitively normal adults. The findings supported the role of gut microbiota in the pathogenesis of cognitive impairment. Further longitudinal follow-up results from this cohort study are needed to elucidate the mechanism underlying how gut microbiota influences the aging brain and contributes to the development of cognitive impairment.

## Data availability statement

The original contributions presented in the study are publicly available. This data can be found at: https://www.ncbi.nlm.nih.gov/sra/, PRJNA937331.

## Ethics statement

The studies involving human participants were reviewed and approved by Far Eastern Memorial Hospital Research Ethics Committee, Institutional Review Board of the National Yang-Ming University. The patients/participants provided their written informed consent to participate in this study.

## Author contributions

YFC and YLC conceived and designed the study. YFC and YCL acquired the data. YPC and YJL assisted in MRI data collection and analysis. KCF and CCL analyzed the gut microbiota data. KCF and YFC interpreted the data and drafted the manuscript. All authors contributed to the manuscript revision and approved the final submitted version.

## Funding

The study was supported by Taiwan Ministry of Science and Technology grant 106-2628-B-010 and 110-2321-B418-001; “Yin Yen-Liang Foundation Development and Construction Plan” of the School of Medicine, National Yang Ming Chiao Tung University Far Eastern Memorial Hospital Joint Research Program 112DN11.

## Conflict of interest

The authors declare that the research was conducted in the absence of any commercial or financial relationships that could be construed as a potential conflict of interest.

## Publisher’s note

All claims expressed in this article are solely those of the authors and do not necessarily represent those of their affiliated organizations, or those of the publisher, the editors and the reviewers. Any product that may be evaluated in this article, or claim that may be made by its manufacturer, is not guaranteed or endorsed by the publisher.

## Footnotes

^1^ https://huttenhower.sph.harvard.edu/galaxy/

^2^ http://surfer.nmr.mgh.harvard.edu/

## References

[ref1] Aitchison J. (1982). The statistical analysis of compositional data. J. R. Stat. Soc. Series B (Method.) 44, 139–160. doi: 10.1111/J.2517-6161.1982.TB01195.X

[ref2] Albert M. S. DeKosky S. T. Dickson D. Dubois B. Feldman H. H. Fox N. C.. (2011). The diagnosis of mild cognitive impairment due to Alzheimer’s disease: recommendations from the National Institute on Aging-Alzheimer’s association workgroups on diagnostic guidelines for Alzheimer’s disease. Alzheimers Dement. 7, 270–279. doi: 10.1016/J.JALZ.2011.03.008, PMID: 21514249 PMC3312027

[ref3] Alkasir R. Li J. Li X. Jin M. Zhu B. (2017). Human gut microbiota: the links with dementia development. Protein Cell 8, 90–102. doi: 10.1007/S13238-016-0338-6, PMID: 27866330 PMC5291774

[ref4] Anderson M. J. (2017). “Permutational multivariate analysis of variance (PERMANOVA)” in Wiley StatsRef: statistics reference online. eds. Balakrishnan N. Colton T. Everitt B. Piegorsch W. Ruggeri F. Teugels J. L. (Chichester: John Wiley & Sons Ltd), 1–15. doi: 10.1002/9781118445112.STAT07841

[ref5] Badri M. Kurtz Z. D. Müller C. L. Bonneau R. (2018). Normalization methods for microbial abundance data strongly affect correlation estimates. bioRxiv:406264. doi: 10.1101/406264

[ref6] Balvočiute M. Huson D. H. (2017). SILVA, RDP, Greengenes, NCBI and OTT – how do these taxonomies compare? BMC Genomics 18, 1–8. doi: 10.1186/S12864-017-3501-4/FIGURES/6 28361695 PMC5374703

[ref7] Berg G. Rybakova D. Fischer D. Cernava T. Vergès M. C. C. Charles T.. (2020). Microbiome definition re-visited: old concepts and new challenges. Microbiome 8, 1–22. doi: 10.1186/S40168-020-00875-0/FIGURES/7 32605663 PMC7329523

[ref8] Bostanciklioğlu M. (2019). The role of gut microbiota in pathogenesis of Alzheimer’s disease. J. Appl. Microbiol. 127, 954–967. doi: 10.1111/JAM.14264 30920075

[ref9] Callahan B. J. McMurdie P. J. Rosen M. J. Han A. W. Johnson A. J. A. Holmes S. P. (2016). DADA2: high resolution sample inference from Illumina amplicon data. Nat. Methods 13, 581–583. doi: 10.1038/NMETH.3869, PMID: 27214047 PMC4927377

[ref10] Cattaneo A. Cattane N. Galluzzi S. Provasi S. Lopizzo N. Festari C.. (2017). Association of brain amyloidosis with pro-inflammatory gut bacterial taxa and peripheral inflammation markers in cognitively impaired elderly. Neurobiol. Aging 49, 60–68. doi: 10.1016/j.neurobiolaging.2016.08.019, PMID: 27776263

[ref11] Chiu Y. L. Tsai H. H. Lai Y. J. Tseng H. Y. Wu Y. W. Peng Y.. (2019). Cognitive impairment in patients with end-stage renal disease: accelerated brain aging? J. Formos. Med. Assoc. 118, 867–875. doi: 10.1016/j.jfma.2019.01.011, PMID: 30744935

[ref12] Chuang Y. F. Liu Y. C. Tseng H. Y. Lin P. X. Li C. Y. Shih M. H.. (2021). Urban-rural differences in the prevalence and correlates of mild cognitive impairment in community-dwelling older adults in Taiwan: the EMCIT study. J. Formos. Med. Assoc. 120, 1749–1757. doi: 10.1016/J.JFMA.2021.03.005, PMID: 33810927

[ref13] Coello K. Hansen T. H. Sørensen N. Munkholm K. Kessing L. V. Pedersen O.. (2019). Gut microbiota composition in patients with newly diagnosed bipolar disorder and their unaffected first-degree relatives. Brain Behav. Immun. 75, 112–118. doi: 10.1016/J.BBI.2018.09.026, PMID: 30261302

[ref14] Coletto E. Latousakis D. Pontifex M. G. Crost E. H. Vaux L. Perez Santamarina E.. (2022). The role of the mucin-glycan foraging *Ruminococcus gnavus* in the communication between the gut and the brain. Gut Microbes 14. doi: 10.1080/19490976.2022.2073784, PMID: 35579971 PMC9122312

[ref15] De Filippo C. Cavalieri D. Di Paola M. Ramazzotti M. Poullet J. B. Massart S.. (2010). Impact of diet in shaping gut microbiota revealed by a comparative study in children from Europe and rural Africa. Proc. Natl. Acad. Sci. U. S. A. 107, 14691–14696. doi: 10.1073/PNAS.1005963107/SUPPL_FILE/PNAS.201005963SI.PDF, PMID: 20679230 PMC2930426

[ref16] Dickerson B. C. Stoub T. R. Shah R. C. Sperling R. A. Killiany R. J. Albert M. S.. (2011). Alzheimer-signature MRI biomarker predicts AD dementia in cognitively normal adults. Neurology 76, 1395–1402. doi: 10.1212/WNL.0B013E3182166E96, PMID: 21490323 PMC3087406

[ref17] Ghosh T. S. Shanahan F. O’Toole P. W. (2022). The gut microbiome as a modulator of healthy ageing. Nat. Rev. Gastroenterol. Hepatol. 19, 565–584. doi: 10.1038/s41575-022-00605-x, PMID: 35468952 PMC9035980

[ref18] Guo M. Peng J. Huang X. Xiao L. Huang F. Zuo Z. (2021). Gut microbiome features of Chinese patients newly diagnosed with Alzheimer’s disease or mild cognitive impairment. J. Alzheimers Dis. 80, 299–310. doi: 10.3233/JAD-201040 33523001

[ref19] Hamidi B. Wallace K. Vasu C. Alekseyenko A. V. (2019). Wd*-test: robust distance-based multivariate analysis of variance. Microbiome 7, 1–9. doi: 10.1186/S40168-019-0659-9/TABLES/3 PMC6444669 30935409

[ref20] Heller A. Coffman S. S. Jarvis K. (2020). Potentially pathogenic calcium oxalate Dihydrate and titanium dioxide crystals in the Alzheimer’s disease entorhinal cortex. J. Alzheimers Dis. 77, 547–550. doi: 10.3233/JAD-200535, PMID: 32804151 PMC7592648

[ref21] Heo J. Seo M. Park H. Lee W. K. Guan L. L. Yoon J.. (2016). Gut microbiota modulated by probiotics and Garcinia cambogia extract correlate with weight gain and adipocyte sizes in high fat-fed mice. Sci. Rep. 6, 1–10. doi: 10.1038/srep33566 27658722 PMC5034228

[ref22] Herd P. Palloni A. Rey F. Dowd J. B. (2018). Social and population health science approaches to understand the human microbiome. Nat Hum Behav 2, 808–815. doi: 10.1038/s41562-018-0452-y, PMID: 31457107 PMC6711373

[ref23] Herlemann D. P. R. Labrenz M. Jürgens K. Bertilsson S. Waniek J. J. Andersson A. F. (2011). Transitions in bacterial communities along the 2000 km salinity gradient of the Baltic Sea. ISME J. 5, 1571–1579. doi: 10.1038/ismej.2011.41, PMID: 21472016 PMC3176514

[ref24] Ho Do M. Seo Y. S. Park H. Y. (2021). Polysaccharides: bowel health and gut microbiota. Crit. Rev. Food Sci. Nutr. 61, 1212–1224. doi: 10.1080/10408398.2020.1755949 32319786

[ref25] Hoyles L. Pontifex M. G. Rodriguez-Ramiro I. Anis-Alavi M. A. Jelane K. S. Snelling T.. (2021). Regulation of blood-brain barrier integrity by microbiome-associated methylamines and cognition by trimethylamine N-oxide. Microbiome 9:235. doi: 10.1186/S40168-021-01181-Z, PMID: 34836554 PMC8626999

[ref26] Hung C. C. Chang C. C. Huang C. W. Nouchi R. Cheng C. H. (2022). Gut microbiota in patients with Alzheimer’s disease spectrum: a systematic review and meta-analysis. Aging 14, 477–496. doi: 10.18632/AGING.203826, PMID: 35027502 PMC8791218

[ref27] Koepsell T. D. Monsell S. E. (2012). Reversion from mild cognitive impairment to normal or near-normal cognition: risk factors and prognosis. Neurology 79, 1591–1598. doi: 10.1212/WNL.0B013E31826E26B7, PMID: 23019264 PMC3475624

[ref28] Kowalski K. Mulak A. (2019). Brain-gut-microbiota axis in Alzheimer’s disease. J Neurogastroenterol Motil 25, 48–60. doi: 10.5056/JNM18087, PMID: 30646475 PMC6326209

[ref29] la Reau A. J. Suen G. (2018). The Ruminococci: key symbionts of the gut ecosystem. J. Microbiol. 56, 199–208. doi: 10.1007/S12275-018-8024-4, PMID: 29492877

[ref30] Li B. He Y. Ma J. Huang P. Du J. Cao L.. (2019). Mild cognitive impairment has similar alterations as Alzheimer’s disease in gut microbiota. Alzheimers Dement. 15, 1357–1366. doi: 10.1016/J.JALZ.2019.07.002, PMID: 31434623

[ref31] Liu P. Jia X. Z. Chen Y. Yu Y. Zhang K. Lin Y. J.. (2021). Gut microbiota interacts with intrinsic brain activity of patients with amnestic mild cognitive impairment. CNS Neurosci. Ther. 27, 163–173. doi: 10.1111/CNS.13451, PMID: 32929861 PMC7816203

[ref32] Liu P. Wu L. Peng G. Han Y. Tang R. Ge J.. (2019). Altered microbiomes distinguish Alzheimer’s disease from amnestic mild cognitive impairment and health in a Chinese cohort. Brain Behav. Immun. 80, 633–643. doi: 10.1016/J.BBI.2019.05.008, PMID: 31063846

[ref33] Marizzoni M. Cattaneo A. Mirabelli P. Festari C. Lopizzo N. Nicolosi V.. (2020). Short-chain fatty acids and lipopolysaccharide as mediators between gut dysbiosis and amyloid pathology in Alzheimer’s disease. J. Alzheimers Dis. 78, 683–697. doi: 10.3233/JAD-200306, PMID: 33074224

[ref34] Martin M. (2011). Cutadapt removes adapter sequences from high-throughput sequencing reads. EMBnet J 17, 10–12. doi: 10.14806/EJ.17.1.200

[ref35] McMurdie P. J. Holmes S. (2013). Phyloseq: an R package for reproducible interactive analysis and graphics of microbiome census data. PLoS One 8:e61217. doi: 10.1371/JOURNAL.PONE.0061217, PMID: 23630581 PMC3632530

[ref36] Morais L. H. Schreiber H. L. Mazmanian S. K. (2020). The gut microbiota–brain axis in behaviour and brain disorders. Nat. Rev. Microbiol. 4, 241–255. doi: 10.1038/s41579-020-00460-0 33093662

[ref37] Nagpal R. Neth B. J. Wang S. Craft S. Yadav H. (2019). Modified Mediterranean-ketogenic diet modulates gut microbiome and short-chain fatty acids in association with Alzheimer’s disease markers in subjects with mild cognitive impairment. EBioMedicine 47, 529–542. doi: 10.1016/J.EBIOM.2019.08.032/ATTACHMENT/AA8D3F9F-49CB-4680-A4B9-DDC4726DD35C/MMC1.PDF 31477562 PMC6796564

[ref39] Ogita T. Yamamoto Y. Mikami A. Shigemori S. Sato T. Shimosato T. (2020). Oral Administration of *Flavonifractor plautii* strongly suppresses Th2 immune responses in mice. Front. Immunol. 11:379. doi: 10.3389/FIMMU.2020.00379/BIBTEX, PMID: 32184789 PMC7058663

[ref40] Pan Q. Li Y. Q. Guo K. Xue M. Gan Y. Wang K.. (2021). Elderly patients with mild cognitive impairment exhibit altered gut microbiota profiles. J Immunol Res 2021, 1–7. doi: 10.1155/2021/5578958, PMID: 34869782 PMC8635943

[ref41] Petersen R. C. (2004). Mild cognitive impairment as a diagnostic entity. J. Intern. Med. 256, 183–194. doi: 10.1111/J.1365-2796.2004.01388.X 15324362

[ref42] Prodan A. Tremaroli V. Brolin H. Zwinderman A. H. Nieuwdorp M. Levin E. (2020). Comparing bioinformatic pipelines for microbial 16S rRNA amplicon sequencing. PLoS One 15:e0227434. doi: 10.1371/JOURNAL.PONE.0227434, PMID: 31945086 PMC6964864

[ref43] Quast C. Pruesse E. Yilmaz P. Gerken J. Schweer T. Yarza P.. (2013). The SILVA ribosomal RNA gene database project: improved data processing and web-based tools. Nucleic Acids Res. 41, D590–D596. doi: 10.1093/NAR/GKS1219, PMID: 23193283 PMC3531112

[ref44] Robin X. Turck N. Hainard A. Tiberti N. Lisacek F. Sanchez J. C.. (2011). pROC: an open-source package for R and S+ to analyze and compare ROC curves. BMC Bioinform 12, 1–8. doi: 10.1186/1471-2105-12-77/TABLES/3 PMC3068975 21414208

[ref45] Saji N. Murotani K. Hisada T. Tsuduki T. Sugimoto T. Kimura A.. (2019a). The relationship between the gut microbiome and mild cognitive impairment in patients without dementia: a cross-sectional study conducted in Japan. Sci. Rep. 9, 1–10. doi: 10.1038/s41598-019-55851-y 31852995 PMC6920432

[ref46] Saji N. Niida S. Murotani K. Hisada T. Tsuduki T. Sugimoto T.. (2019b). Analysis of the relationship between the gut microbiome and dementia: a cross-sectional study conducted in Japan. Sci. Rep. 9:1008. doi: 10.1038/s41598-018-38218-7, PMID: 30700769 PMC6353871

[ref47] Scheltens P. de Strooper B. Kivipelto M. Holstege H. Chételat G. Teunissen C. E.. (2021). Alzheimer’s disease. Lancet 397, 1577–1590. doi: 10.1016/S0140-6736(20)32205-4/ATTACHMENT/C76D9418-879C-4AD6-993B-BE3AFE02F0F6/MMC1.PDF, PMID: 33667416 PMC8354300

[ref48] Segata N. Izard J. Waldron L. Gevers D. Miropolsky L. Garrett W. S.. (2011). Metagenomic biomarker discovery and explanation. Genome Biol. 12, 1–18. doi: 10.1186/GB-2011-12-6-R60/FIGURES/6 PMC3218848 21702898

[ref49] Sheng C. Lin L. Lin H. Wang X. Han Y. Liu S. L. (2021). Altered gut microbiota in adults with subjective cognitive decline: the SILCODE study. J. Alzheimers Dis. 82, 513–526. doi: 10.3233/JAD-210259, PMID: 34024839

[ref50] Sheng C. Yang K. He B. Du W. Cai Y. Han Y. (2022). Combination of gut microbiota and plasma amyloid-β as a potential index for identifying preclinical Alzheimer’s disease: a cross-sectional analysis from the SILCODE study. Alzheimers Res. Ther. 14, 1–15. doi: 10.1186/S13195-022-00977-X/FIGURES/5 35164860 PMC8843023

[ref51] Shimada H. Doi T. Lee S. Makizako H. (2019). Reversible predictors of reversion from mild cognitive impairment to normal cognition: a 4-year longitudinal study. Alzheimers Res. Ther. 11, 1–9. doi: 10.1186/S13195-019-0480-5/TABLES/4 30867057 PMC6416893

[ref52] Sun Y. Ho C. T. Zhang Y. Hong M. Zhang X. (2023). Plant polysaccharides utilized by gut microbiota: new players in ameliorating cognitive impairment. J. Tradit. Complement. Med. 13, 128–134. doi: 10.1016/J.JTCME.2022.01.003, PMID: 36970456 PMC10037067

[ref53] Verhaar B. J. H. Hendriksen H. M. A. de Leeuw F. A. Doorduijn A. S. van Leeuwenstijn M. Teunissen C. E.. (2022). Gut microbiota composition is related to AD pathology. Front. Immunol. 12:6030. doi: 10.3389/FIMMU.2021.794519/BIBTEX PMC8843078 35173707

[ref54] Vogt N. M. Kerby R. L. Dill-McFarland K. A. Harding S. J. Merluzzi A. P. Johnson S. C.. (2017). Gut microbiome alterations in Alzheimer’s disease. Sci. Rep. 7:13537. doi: 10.1038/s41598-017-13601-y, PMID: 29051531 PMC5648830

[ref55] Wanapaisan P. Chuansangeam M. Nopnipa S. Mathuranyanon R. Nonthabenjawan N. Ngamsombat C.. (2022). Association between gut microbiota with mild cognitive impairment and Alzheimer’s disease in a Thai population. Neurodegener Dis 22, 43–54. doi: 10.1159/000526947 36070704

[ref56] Xiao C. Fedirko V. Beitler J. Bai J. Peng G. Zhou C.. (2021). The role of the gut microbiome in cancer-related fatigue: pilot study on epigenetic mechanisms. Support. Care Cancer 29, 3173–3182. doi: 10.1007/S00520-020-05820-3/TABLES/3, PMID: 33078326 PMC8055716

[ref57] Yıldırım S. Nalbantoğlu Ö. U. Bayraktar A. Ercan F. B. Gündoğdu A. Velioğlu H. A.. (2022). Stratification of the gut microbiota composition landscape across the Alzheimer’s disease continuum in a Turkish cohort. mSystems 7. doi: 10.1128/MSYSTEMS.00004-22/SUPPL_FILE/MSYSTEMS.00004-22-SF005.PDF PMC8823292 35133187

[ref58] Zhang X. Wang Y. Liu W. Wang T. Wang L. Hao L.. (2021). Diet quality, gut microbiota, and microRNAs associated with mild cognitive impairment in middle-aged and elderly Chinese population. Am. J. Clin. Nutr. 114, 429–440. doi: 10.1093/AJCN/NQAB078, PMID: 33871591

[ref59] Zhuang Z. Q. Shen L. L. Li W. W. Fu X. Zeng F. Gui L.. (2018). Gut microbiota is altered in patients with Alzheimer’s disease. J. Alzheimers Dis. 63, 1337–1346. doi: 10.3233/JAD-180176 29758946

