# Supplementary material for: Altered gut microbiota in older adults with mild cognitive impairment: a case-control study
Source: Front Aging Neurosci. 2023 May 25;15:1162057. doi: 10.3389/fnagi.2023.1162057 (PMC10281289; doi:10.3389/fnagi.2023.1162057)
Supplement: Supplementary file 1 [file Table_1.pdf]

Supplementary Material

Altered Gut Microbiota in Older Adults with Mild Cognitive Impairment: a Case-Control study

Kang-Chen Fan, Chen-Ching Lin, Yi-Chien Liu, Yi-Ping Chao, Yen-Jun Lai, Yen-Ling Chiu, Yi-Fang Chuang*

*** Correspondence:** Yi-Fang Chuang: chuangy@nycu.edu.tw

1 Supplementary Figures and Tables

1.1 Supplementary Tables

		MCI n=31		Normal n=65	
	Age	Male	Female	Male	Female
Urban	<75	7	5	12	12
	≥75	5	3	7	12
Rural	<75	3	5	5	6
	≥75	1	2	7	4

Supplementary Table 1. The exact numbers of cases and controls in each stratum.

Reads	Input	Filtered	%	Denoised Forward	%	Denoised Reverse	%	Merged	%	Nonchimeric	%
MCI	155109.8	117792.3	77.5	116701.8	76.7	116793.4	76.8	111374.5	73.1	99771.4	65.5
Normal	122941.7	96982.6	80.5	95980.8	79.6	96087.4	79.7	91025.8	75.4	81194.4	67.4
Total	133329.3	103702.4	79.5	102671.9	78.7	102773.7	78.8	97596.7	74.7	87193.2	66.8

Supplementary Table 2. DADA2 denoising process of 16S rRNA reads.

	MCI					
	Model 1*		Model 2†		Model3‡	
	ß (SE)	p-value	ß (SE)	p-value	ß (SE)	p-value
Richness indices						
Observed	-6.1 (16.6)	0.71	-7.5 (15.3)	0.62	-2.2 (16.4)	0.89
Chao1	-5.8 (16.7)	0.73	-7.3 (15.4)	0.64	-2.2 (16.6)	0.89
α-diversity indices						
Shannon	-0.1 (0.1)	0.33	-0.1 (0.1)	0.30	-0.05 (0.1)	0.65
Simpson	-0.003 (0.007)	0.62	-0.003 (0.007)	0.65	0.0004 (0.0009)	0.95
InvSimpson	-3.3 (2.9)	0.26	-3.3 (2.8)	0.24	-2.7 (3.0)	0.36

* Model 1: unadjusted

† Model 2: adjusted for age, gender, community (urban and rural)

‡ Model 3: adjusted for age, gender, community (urban and rural), education level (≤9 and >9 years), hypertension

Supplementary Table 3. The association between MCI and richness / α-diversity indices.

	PERMANOVA						Wd*	
	Df	Distance Method	Sum Sq	R^2	Pseudo-F	p-value	Statistic	p-value
MCI	1	Bray-Curtis	0.317	0.011	1.089	0.25	1.07	0.25
		Jaccard	0.390	0.011	1.046	0.23	1.04	0.28
		UniFrac	0.414	0.018	1.709	**0.006**	1.57	**0.02**
		Weighted UniFrac	0.020	0.010	0.921	0.47	0.79	0.68

Supplementary Table 4. PERMANOVA and Wd* test of beta-diversity

Volume, cm^3, mean ± SD	MCI n = 26	Normal n = 64	p-value
Total brain	999.2 ± 131.1	1035.0 ± 98.3	0.16
Ventricle	35.5 ± 11.8	32.5 ± 12.4	0.29
Gray matter	407.9 ± 48.2	422.8 ± 39.9	0.13
White matter	411.0 ± 66.7	431.5 ± 53.9	0.13
Hippocampus	7.5 ± 1.0	7.8 ± 0.8	0.15
Entorhinal	3.5 ± 0.9	3.7 ± 0.6	0.36
Amygdala	2.9 ± 0.5	3.0 ± 0.4	0.38

Thickness, cm, mean ± SD	MCI n = 26	Normal n = 64	p-value
AD-score	2.5 ± 0.15	2.5 ± 0.11	0.53

Supplementary Table 5. Characteristics of brain structure between MCIs and cognitively normal adults.

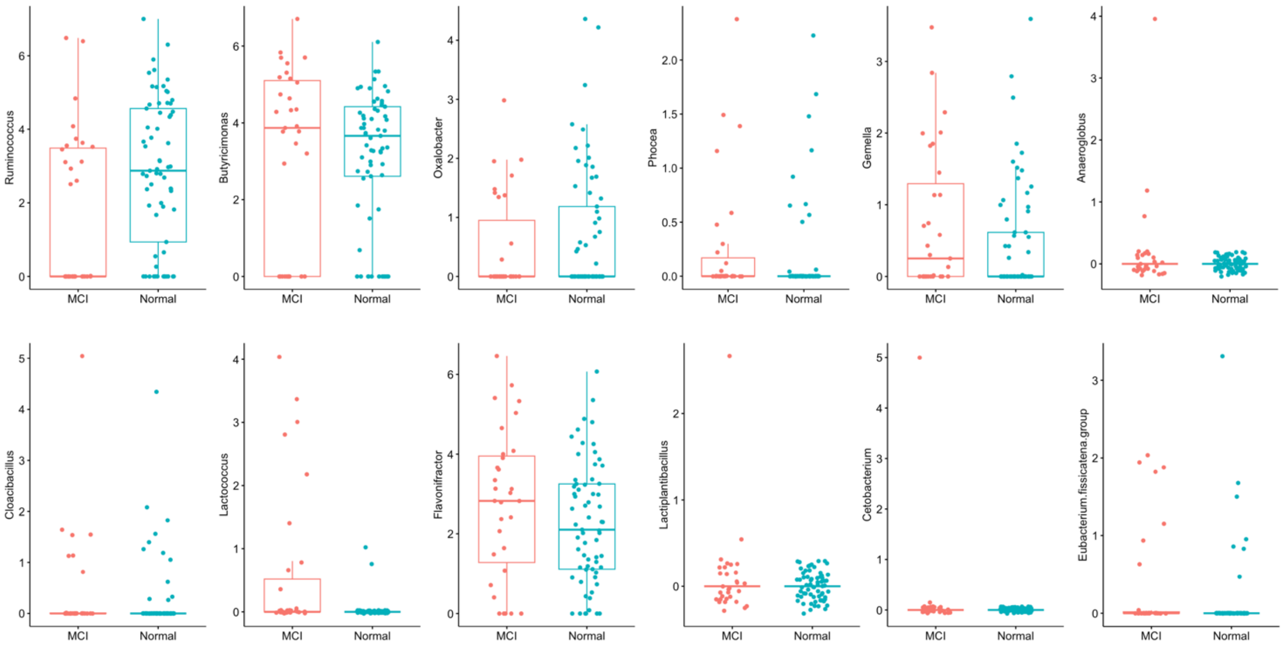

Supplementary Figure 1. Centred-log ratio (CLR) transformed relative abundances of genera different between MCIs and cognitively normal adults, which were identified by linear discriminant analysis (LDA) effect size (LEfSe).
